# Strategies to overcome the main challenges of the use of CRISPR/Cas9 as a replacement for cancer therapy

**DOI:** 10.1186/s12943-021-01487-4

**Published:** 2022-03-03

**Authors:** Mohammed Fatih Rasul, Bashdar Mahmud Hussen, Abbas Salihi, Bnar Saleh Ismael, Paywast Jamal Jalal, Anna Zanichelli, Elena Jamali, Aria Baniahmad, Soudeh Ghafouri-Fard, Abbas Basiri, Mohammad Taheri

**Affiliations:** 1 Department of Medical Analysis, Faculty of Applied Science, Tishk International University, Erbil, Kurdistan Region Iraq; 2 Department of Pharmacognosy, College of Pharmacy, Hawler Medical University, Kurdistan region, Erbil, Iraq; 3 Center of Research and Strategic Studies, Lebanese French University, Erbil, Iraq; 4 Department of Biology, College of Science, Salahaddin University-Erbil, Erbil, Iraq; 5 Department of Pharmacology and Toxicology, College of Pharmacy, Hawler Medical University, Kurdistan region, Erbil, Iraq; 6 Biology Department, College of Science, University of Sulaimani, Sulaimani, Iraq; 7 Department of Biomedical Sciences, University of Westminster, London, UK; 8 Department of Pathology, Loghman Hakim Hospital, Shahid Beheshti University of Medical Sciences, Tehran, Iran; 9 Institute of Human Genetics, Jena University Hospital, Jena, Germany; 10 Department of Medical Genetics, School of Medicine, Shahid Beheshti University of Medical Sciences, Tehran, Iran; 11 Urology and Nephrology Research Center, Shahid Beheshti University of Medical Sciences, Tehran, Iran; 12 Men’s Health and Reproductive Health Research Center, Shahid Beheshti University of Medical Sciences, Tehran, Iran

**Keywords:** CRISPR, Cas9, Cancer therapy, Gene editing, Gene modification challenges

## Abstract

CRISPR/Cas9 (clustered regularly interspaced short palindromic repeats-associated protein 9) shows the opportunity to treat a diverse array of untreated various genetic and complicated disorders. Therapeutic genome editing processes that target disease-causing genes or mutant genes have been greatly accelerated in recent years as a consequence of improvements in sequence-specific nuclease technology. However, the therapeutic promise of genome editing has yet to be explored entirely, many challenges persist that increase the risk of further mutations. Here, we highlighted the main challenges facing CRISPR/Cas9-based treatments and proposed strategies to overcome these limitations, for further enhancing this revolutionary novel therapeutics to improve long-term treatment outcome human health.

## Background

Cancer is one of the leading causes of disease-related death, increasing worldwide incidence [1]. At the same time, advancements have been achieved in the prevention and therapeutic approaches, resulting in longer lifetimes or even cures for certain patients with cancer. Unfortunately, chemotherapy and radiotherapy, the two gold stones in cancer treatment, are also painful for patients and cause severe side effects [2]. Therefore, developing innovative anti-cancer therapies with less side effects needs a comprehensive understanding of cancer biology. The most recent advancements in sequencing technology have made it possible to study the cancer genome more effectively and at a lower cost than ever before. The use of an integrated strategy that incorporates genomic and transcriptomic advancements can provide a comprehensive view of an individual’s genome. Additionally, this method is used to make valuable decisions relating to patient therapeutic options [3].

Different genomic engineering tools have been performed in cancer therapy such as ZFNs and TALENs by targeting DNA domain-binding proteins. Still, their efficacy was limited due to the inability to target epigenetic modification that arises in tumorigenesis [4]. Recently, a more flexible genome editing technique, CRISPRs linked with HNH domain protein Cas9, promises efficient, long-term safety cancer treatment [5]. The CRISPR/Cas9 system, unlike previous genome editing methods that used protein-DNA interactions to mediate sequence recognition, uses an RNA molecule to mediate binding. CRISPR loci, which are made up of alternating repeat-spacer units, and CRISPR-associated (Cas) proteins, are derived from a prokaryotic host defense system that protects against viral genomes and plasmids [6]. Based on the method of recognition and cleavage, CRISPR/Cas systems are divided into two classes, which are further divided into six types and various subtypes [7]. Class 1 systems cleave with protein complexes, whereas Class 2 systems only cleave with one protein, creating an opportunity for genome engineering [8]. However, certain targeting limitations apply to all Class 2 systems (types II, V, and VI). For example, a protospacer flanking sequence is recognized by Type VI systems, which use Cas13 to cleave RNA [9]. In addition, type II and Type V systems recognize the adjacent protospacer motif (PAM), a conserved 2–5 bp sequence [10]. For example, the Cas12a/Cpf1 protein uses a simple crRNA and recognizes a PAM directly before the protospacer, such as T-rich PAMs (TTTN) [11]. Conversely, type II Cas9 nuclease recognizes PAM sequences downstream of the protospacer [12]. The most well-characterized and broadly applied CRISPR system is the type II CRISPR/Cas9 system.

**Fig. 1:**
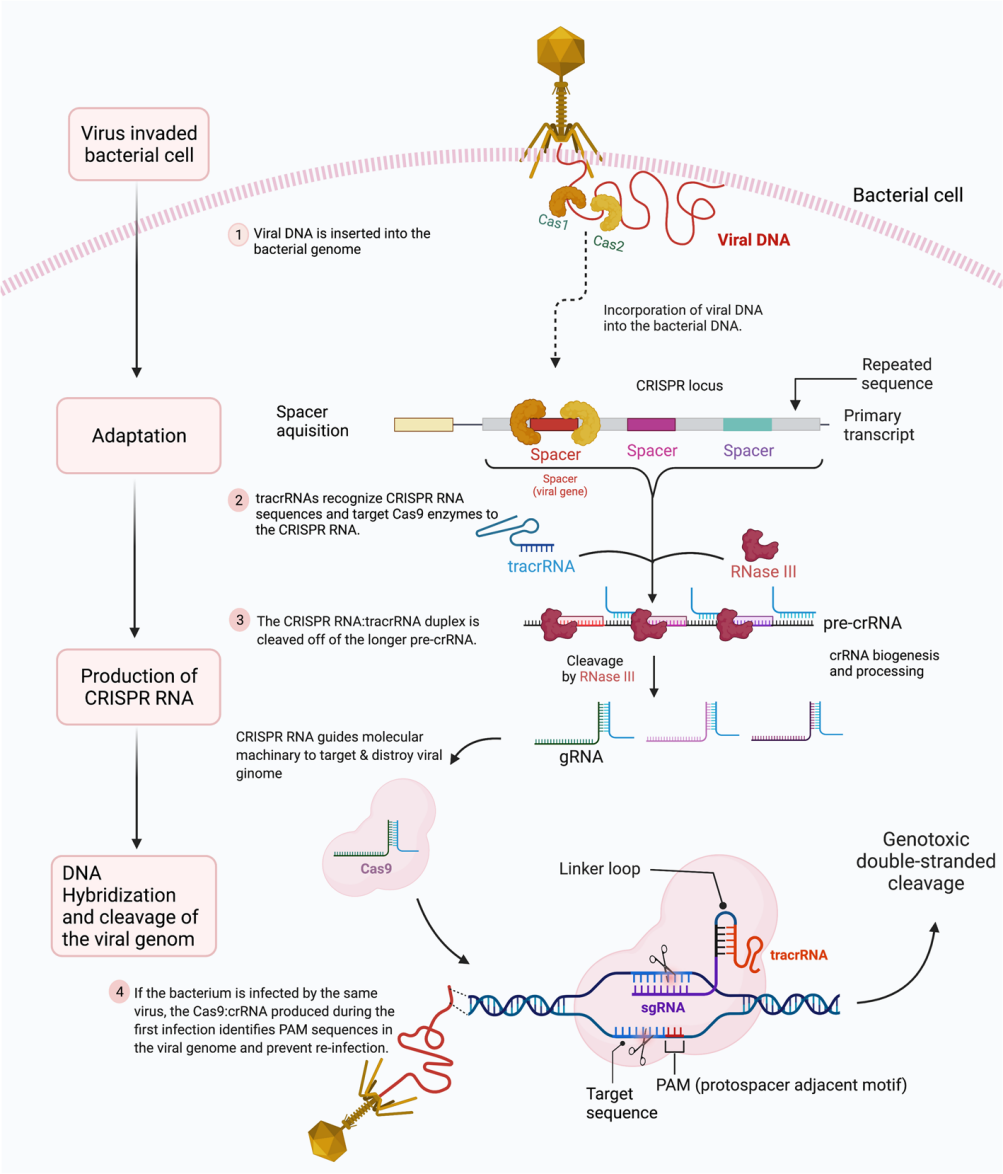
The stages of CRISPR/Cas adaptive immunity. The three phases of the CRISPR/Cas9 system are depicted schematically. When phage DNA is injected into a bacterial cell, the Cas1–Cas2 adaptation module proteins are activated, which remove spacer-sized segments of phage DNA and channel them into the CRISPR array. The CRISPR array is transcribed, and the resulting pre-crRNA is processed at repeat sequences to form crRNAs during CRISPR RNA biogenesis. The Cas protein effectors bind individual crRNAs. Effectors programmed by suitable crRNA attach to phage DNA with sequences matching a CRISPR spacer in the cell, and the resulting R-loop complex is destroyed by Cas executor nuclease

Cas9 is an RNA-guided endonuclease that recognizes and cleaves target DNAs that have template strand pairing to the guide RNA, and it requires RNA molecule known as the trans-activating crRNA (tracrRNA). TracrRNA promotes crRNA binding and processing. Moreover, a linker can join the tracrRNA and crRNA into a single molecule known as the single guide RNA used in genome editing (sgRNA) (Fig. 1).

Cas9 is an RNA-guided endonuclease that recognizes and cleaves target DNA that have template strand pairing to the guide RNA, which is composed of Crispr RNA (crRNA) and tracrRNA [13]. crRNA, which has a [17–20] nucleotide sequence that is complementary to the target DNA, and tracrRNA, which acts as a Cas nuclease binding scaffold [14].

The CRISPR/Cas9 system has been successfully applied to *in vitro* cancer research by inhibiting one or more oncogenic molecular pathways (Table 1). However, the *in vivo* use of the CRISPR/Cas9 system has faced many challenges such as the occurrence of off-targeting modifications, the possibility of causing autoimmune diseases, the identification of a proper delivery technique, and, lastly, ethical concerns. As a result, research scientists follow different procedures and investigate various bioinformatics tools to prevent, or at least reduce, these obstacles to make the CRISPR/Cas9 system more suitable for treating cancer in the human body. This review summarizes some of the main limitations of using CRISPR/Cas9 in clinical trials and some of the strategies applied in previous studies to overcome these limitations. Hopefully, this study provides a comprehensive overview of the main roadblocks to implementing this promising technique *in vivo*, helping future researchers focus their efforts on tackling them and making CRISPR come alive as a powerful strategy to treat cancer.

### Innovative advances in CRISPR/Cas9 gene-editing technology

**Fig. 2:**
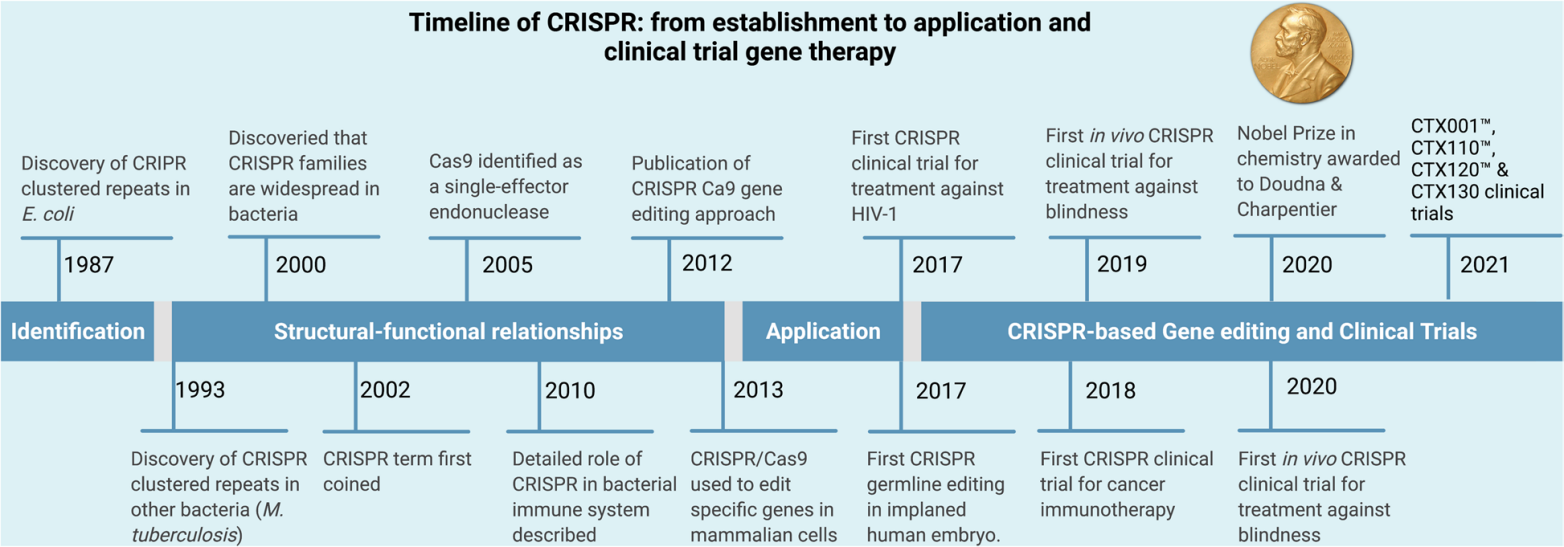
Timeline highlighting main events of identification, CRISPR development (structural-functional relationships), applications, and CRISPR-based gene editing and clinical trials

When Japanese scientists found several previously undiscovered tandem repeats in the *E. coli* genome in 1987, they didn’t report the biological relevance of those findings [15]. However, the role of these sequences remained unknown until they were termed Clustered Regularly Interspaced Short Palindromic Repeats (CRISPR) in 2002 [16]. Then, in 2005, the CRISPR loci were shown to play a significant role in adaptive immunity by three different study teams [17–19]. In 2007, Barrangou and his team revealed that viral gene sequences integrated by bacteria might modify the bacterium’s resistance to phages [20]. Brouns et al. in 2008 discovered that non-coding RNA produced from the CRISPR incorporating short fragments might direct the CRISPR-associated (Cas) proteins to the target-specific portion of DNA, allowing it to perform a protective function [21]. Deltcheva et al. discovered that trans-coding crRNA (tracrRNA) was related to the maturation and processing of pre-crRNA, and their research revealed new destinations for crRNA development [22]. *In vivo* studies in 2012 showed that mature crRNA produced two unique RNA structures when base-paired with tracrRNA, guiding CRISPR-associated protein Cas9 to create double-stranded (ds) DNA cleavage [23]. Subsequently, Cong and Mali teams made genome editing with the CRISPR/Cas9 system possible, who used two different type II Cas systems to make DNA cuts in cell cultures [24, 25]. Once the CRISPR/Cas9 technology was developed, many CRISPR/Cas9-based tools for gene editing at the DNA and RNA levels were created by 2020, with fast advancements in the technology since [26, 27] (Fig. 2).

### Overview of CRISPR/Cas9-based genome editing

CRISPR is a response of the bacterial and archaea immune system to protect themselves from virus infections [28]. Approximately half of the bacteria have a CRISPR/Cas system [29, 30] a defense mechanism that allows the bacterial cell to memorize, recognize and beat recurrently infecting agents [31]. In this system, short guide CRISPR RNAs (crRNA) interfere with invading nucleic acids in a sequence-specific manner. CRISPR/Cas is composed of a genomic locus termed CRISPR that contains harsh repeating elements separated by unique sequences (spacers), which derive from Mobile genetic factors like phages, plasmids, or transposons. An AT-rich region is typically found at the beginning of Cas genes, which encode Cas proteins [32]. Nowadays, according to the structure and function of the Cas protein, the CRISPR/Cas systems can be divided into two classes (class I, class II), which are further categorized into six types (type I–VI) [33]. Class I consists of multiprotein complexes responsible for the cleavage of nucleic acid.

In contrast, in class II, only a single protein, Cas9, is used to read, identify and cleave the DNA target sequence [33]. In CRISPR technology, a single protein method is more effective than a multiprotein approach, hence the class II system is more often used, especially in research [10]. Figure 1 illustrates the details of the Type II CRISPR/Cas9 system. For instance, deactivated Cas9 can be utilized to target the epigenome by inhibiting the enzymatic activity of HNH domains without causing sequence disruption [34]. The guide RNA is composed of two core parts; the first is required to bind the RNA to the Cas protein, and the second part, called a spacer, consists of about 20 nucleotides and is responsible for identifying and binding to the targeted site [35]. Furthermore, the PAM sequence is a short DNA sequence usually between 2 and 6 nucleotides that is also required to identify the exact target site on the DNA, and it is located three base pairs from the site where the DNA will be cut, and the mutation will be introduced [10] (Fig. 1).

### Anticancer application of CRISPR/Cas9 gene editing and clinical trials

**Fig. 3:**
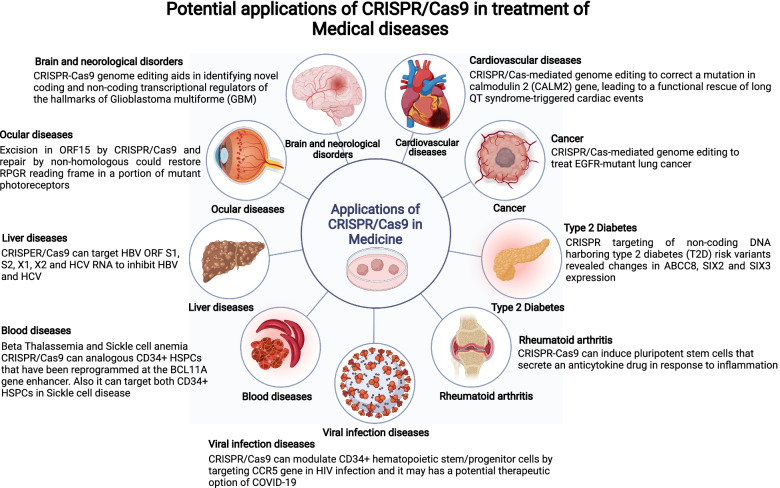
CRISPR/Cas9-mediated treatment has the potential to cure a variety of diseases. The number of diseases that CRISPR is now used to treat is rising by the day. The CRISPR/Cas9 system has been used to generate many disease-based models for many important human diseases, including viral diseases, neurological diseases, cancer, ocular disease, blood diseases, and cardiovascular diseases and disorders, as well as other complex genetic human diseases, according to data from clinical trials released recently

Cancer initiation and spread are mediated by mutations and dysregulation of a variety of genes [36] such as oncogenes, tumor suppressor genes, and stem cell-associated genes, chemo-resistant genes and metabolic genes. Cancer treatment’s primary goal is to halt cancer cell growth and development by repairing mutations and restoring dysregulated gene expression. Since its inception, the CRISPR/Cas9 gene-editing method has been widely used in cancer research, with promising results. Georgiadis et al. recently demonstrated that fratricide-resistant T cells can be generated by removing and replacing the TCR/CD3 and CD7 with lentiviral-mediated production of CARs specific for the CD3 or CD7[37]. Table 1 lists some of the target genes, tumors, and studies that show the effectiveness of CRISPR/Cas9 in correcting these alterations. Based on promising pre-clinical results, the CRISPR/Cas9 system can be used in clinical settings to target cancer-causing genes (Fig. 3). The efficacy of CRISPR-based cancer therapeutics is now being investigated in a number of clinical trials (Table 2).

The programmed cell death-1 (PD-1) protein expression is being targeted by several of these clinical studies. For example, a monoclonal antibody against PD-1 called pembrolizumab exhibits anti-tumor activity in Non-Small Cell Lung Cancer (NSCLC), suppressing the immune system’s ability to produce PD-1 and PD-L1 (programmed death-ligand 1), dramatically improves patients’ survival rate [38]. Because the FDA has approved PD-1 inhibitors for cancer immunotherapy, PD-1 is an intriguing target for immunotherapy. In addition, CRISPR/Cas9 has been used in patients to begin targeting PD-1 (NCT02793856). They used CRISPR/Cas9 to suppress PD-1 expression in metastatic cells from NSCLC patients. The cells were cultured and modified before being reintroduced into the patient [39].

PD-1 knockout-engineered immune cells to treat metastatic NSCLC will be tested for safety in a dosages trial. Additional trials targeting PD-1 expression in T-cells are currently done in other types of cancer such as renal, bladder, and prostate cell malignancies [40]. Similarly, PD-1 deletion has been used in T-cells in phase II clinical trials for esophagus cancer (NCT03081715). Furthermore, the ability of CRISPR gene editing for cancer immunotherapy to persist for up to 9 months, suggests that immunogenicity is low under these settings and demonstrates the practicality of CRISPR gene editing for cancer immunotherapy [41]. Clinical experiments are also using CRISPR/Cas9 to create chimeric antigen receptor (CAR) T cells.

**Fig. 4:**
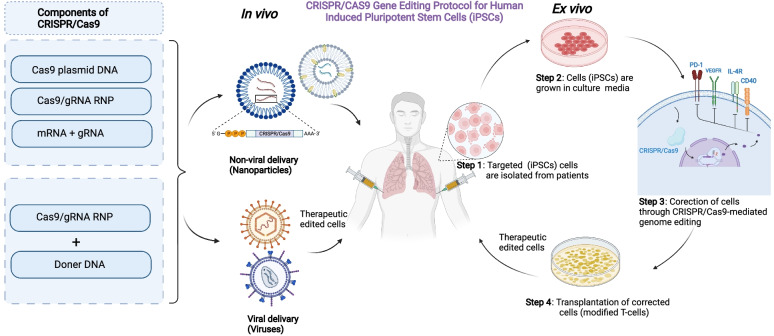
Overview of CRISPR/Cas9-based gene editing of human iPSCs which includes both *in vivo* and *in vitro* methods. Gene editing techniques like CRISPR/Cas9 have allowed researchers to develop isogenic control human iPS cell lines to study the genetic pathways underlying disease and cellular function

The first-in-human trial was conducted by scientists from the university of Pennsylvania applying CRISPR/Cas9 genome-edited NY-ESO-1 TCR cells for cancer patients [42] including advanced multiple myeloma (MM) myxoid/round cell liposarcoma (MRCL), and synovial sarcoma (NCT03399448). They showed that T cells were proven to be safe, viable, and long-lasting [42]. Furthermore, using CRISPR to eliminate endogenous TCR and PD-1 might improve tumor rejection activity [40]. Additionally, the allogeneic CAR T-cells targeted to the CD19 antigen were produced by combining the lentivirus-delivered CAR receptors and electroporation-delivered CRISPR RNA to alter the natural TCR and B2M genes. For patients with leukemia, this strategy may help avoid the host’s immune system and hence avoid graft-versus-host-disease complications. Consequently, additional CRISPR clinical trials (phase III) used CRISPR-edited CAR T-cells with dual specificity for CD19 and CD20/CD22, which can identify and destroy CD19-negative malignant cells by identification of CD20/CD22 (NCT03398967). This may have been a helpful adjunctive treatment for an extensive range of the population. In another work, Chen et al. applied CRISPR/Cas13a to disrupt human papillomavirus16/18 E6/E7 mRNAs using an emerging programmed CRISPR technology. They revealed that HPV 16/18 E6/E7 mRNA was successfully and selectively knocked down using a modified CRISPR/Cas13a system, causing growth suppression and cell death in HPV 16 and 18 positive SiHa and HeLa cell lines, but not in the HPV -negative C33A cells [43]. An additional CRISPR clinical study has been planned to test new medications and determine their effectiveness (NCT03332030). In this study, patients with Neurofibromatosis type 1 (NF1) were used to create an induced pluripotent stem cell bank (iPSC) (NF1). NF1 is a common neurocutaneous disease that frequently develops tumors of both benign and malignant types [44]. The main method that used in vivo and in vitro CRISPR/Cas9 study to treat diseases showed in Figs. 3 and 4.

To identify a particular target drug for NF1, CRISPR/Cas9 was used to create NF1 homozygous (NF1-/-) and NF1 heterozygous (NF1+/-) cell lines, as well as NF1 wild type (NF1+/+). The discovery of NF1-targeted therapies may be aided by the opposite or alleviated characteristics. Despite promising clinical trial results, more research is needed to ensure that CRISPR/Cas9 is a safe and effective method of treating human cancers [45]. On the other hand, CRISPR Cas9 indirectly can be used in cancer therapy to find out the drug-resistance mutation in a short period of time. For example, through applying CRISPR Cas9, only in 40 min can determine the FLT3-F691L with a sensitivity of 0.1% [46].

**Table 1:** list of genes that were knocked out using CRISPR/Cas9 technique in different types of cancer and their effects

Types of Cancer	Target Gene	Cell line	Animal model	Mode of action	Delivery Method	Function	Ref
Breast Cancer	P53, PTEN, RB1, NF1	-	Mice	Knockout	lentiviruses	For both endocrine and chemotherapy, mutated organoids had a greater response rate for mutated organoids.	[47]
Breast cancer	miR-23b and miR-27b	MCF7	Mice	Knockout	lentiviruses	miR-23b and miR-27b have been shown to be oncogenic miRs, and miR-27b reduces tumor development after knockout.	[48]
Breast cancer	PTEN, AKT1, PIK3CA	NIH3T3	Mice	Knockout	lentiviruses	we show that somatic base editing is feasible and effective at installing defined missense and nonsense mutations at endogenous loci in a mouse model of TNBC. we show that somatic base editing is possible and effective at installing defined missense and nonsense mutations at endogenous loci in a mouse model of TNBC. we show that somatic base editing is feasible and effective at installing defined missense and nonsense mutations at endogenous loci in a mouse model of TNBC. we show that somatic base editing is feasible and effective at installing defined missense and nonsense mutations at endogenous loci in a mouse model of TNBC. we show that somatic base editing is feasible and effective at installing defined missense and nonsense mutations at endogenous loci in a mouse model of TNBC. we show that somatic base editing is feasible and effective at installing defined missense and nonsense mutations at endogenous loci in a mouse model of TNBC. In a TNBC mouse model, somatic base editing may effectively introduce specified missense and nonsense mutations.	[49]
Breast cancer	CBEs	HEK293-T, MDA-MB-231, MCF-7	-	Knockout	lentiviruses	For ER-driven breast cancer cell growth, unique CTCF-mediated chromatin configurations are required.	[50]
Breast cancer	AURKA	HEK293T, MDA-MB-231, SKBR3, MCF7	-	Knockout	lentiviruses	CHR-6494 might be used in conjunction with MLN8237 to enhance its anti-cancer benefits.	[51]
Breast cancer	CXCR4 and CXCR7	MDA-MB-231	-	Knockout	lentiviruses	The knockout of CXCR4 and CXCR7 genes reduces the binding ability and activities of CXCL12, slows the growth of TNBC cells, and may be used to treat TNBC.	[52]
Breast cancer	PARP1	MDA-MB-231, MDA-MB-436	-	Knockout		The effectiveness of PARP1 inhibition with chemotherapy for TNBC treatment varies.	[53]
Breast cancer	BRCA1	MDA-MB-231, ASC	-	Knockdown	lentiCRISPRv2 vector	Breast cancer development is promoted by BRCA1 mutation in the tumor microenvironment.	[54]
Breast cancer	APOBEC3G	MCF10A and HCC1806	-	Knockout	lipofection	multiple clones evaluated for APOBEC3G gene knockout success.	[55]
Breast cancer	CDK4, SRPK1, DNMT1	MCF10A, HEK 293T and GP2-293	Mice	Knockout	lentiviruses	Transcriptional epistasis influences around 50% of differentially expressed genes in cancer cells.	[56]
Breast cancer	CDH1	MCF-7	Rats	Knockout	Plasmid Transfection	It is possible to target cancer-related genes using any genome editing technique.	[57]
Breast Cancer	OPN	MDA-MB-231	-	Knockout	CaCl2 transformation	Inactivating osteopontin with CRISPR/Cas9 may overcome radioresistance in breast cancer.	[58]
Breast Cancer	BRCA	MDA-MB-231	-	Knockout	lentivirus	Targeting a group of genes offers new possibilities for PARPi combination treatments.	[59]
Breast Cancer	TMEM106A	MDA-MB-231, MDA-MB-468	-	Knockout	-	In breast cancer, TMEM106A inhibits WDR77 translocation.	[60]
Breast and Lung cancer	CDK4, p107, TGFβ1	A549 and MCF7	-	Knockout	-	After being challenged with CRISPR cassettes, both cell lines showed a considerable decline in cell count.	[61]
Lung cancer	PKP2	H1299, A549, H460	-	Knockout	-	Methylation of PKP2 plays an essential factor in radioresistance by stabilizing catenin by CRISPR/Cas9 library screening.	[62]
Lung Cancer	Trp53, KRas	HEK-293T	Adult Mice	Knockout	lentiviruses	Using the CRISPR toolset, researchers may rapidly build novel, therapeutically relevant alternative models for biomedical research.	[63]
Colon Cancer	KRAS	HT29, WIDR, HCT116, LS174T, and HEK293T; SW480 and A549; and CFPAC-1	-	Knockout	two-vector lentivirus system	GRB7-PLK1 has a critical axis for RTK tolerance. PLK1 and thus a suitable target for synergizing MEK inhibitors in CRC patients with KRAS mutations.	[64]
Colon Cancer	Klotho	Caco-2	-	Knockout	-	By causing apoptosis, Klotho gene overexpression in Caco-2 cells by CRISPR/Cas9 inhibits cell growth.	[65]
Colon cancer	uPAR	CRL1619, CCL247	-	Knockout	Okayama-Berg vector	Knockout of the uPAR gene Leads to tumor growth inhibition, EGFR downregulation, and an increase in stemness markers.	[66]
Prostate cancer	Tceal1	Mouse: SP1 Human: PC3M, LNCaP, DU145, CWR22, RWPE	-	Knockdown	lentivirus	TCEAL1 deletion causes a different cell cycle profile than docetaxel alone, with more subG1 cell death and polyploidy.	[67]
Prostate cancer	miRNA (miR)205, miR221, miR222, miR30c, miR224, miR4553, miR23b, miR505	LNCaP	-	Knockout	Lentivirus	Functional classification of prostate cancer-associated miRNAs through CRISPR/Cas9 mediated gene knockout	[68]
Prostate cancer	BRAF	CWR-R1	-	Knockout	lentiviral	MAPK/AR co-targeting may help patients with active MAPK pathways, especially those with oncogenic BRAF mutations.	[69]
Prostate cancer	TP53	PC-3	-	Knock-in	lentiviral	The impact of CRISPR/Cas9 guided mutant TP53 gene repair in PC-3 human prostate cancer cells	[70]
Prostate cancer	ECE1, ABCA12, BPY2, EEF1A1, RAD9A, and NIPSNAP1	DU145 and PC3	-	Knockdown	lentiviral	Prostate cancer metformin Resistance related gene screening using CRISPR-Cas9.	[71]
Ovarian cancer	EGFL6	SKOV3	-	Knockout	Lentivirus	EGFL6 knockout by CRISPR/Cas9 inhibited tumor angiogenesis.	[72]
Ovarian cancer	ZNF587B and SULF1	A2780, SKOV3, IOSE80	-	Knockout	Lentivirus	Based on genome-scale CRISPR/Cas9 screening, loss of ZNF587B and SULF1 led to cisplatin resistance.	[73]
Ovarian cancer	AR and Nanog expression	A2780, SKOV3	-	Knock in	Lentivirus	Nanog interaction with androgen receptor signaling axis regulates ovarian cancer stem cells using CRISPR/Cas9.	[74]
Ovarian cancer	ITK	SKOV3	Human	Knockout	Lentivirus	For ovarian cancer metastasis, ITK (IL2 Inducible T Cell Kinase) may be a possible cancer suppressor gene.	[75]
thyroid cancer	AXIN1	ACT-1	-	Knockout	Viral vector	CRISPR/Cas9 has been used to effectively create an ACT-1 undifferentiated thyroid cancer cell line lacking the AXIN1 gene.	[76]
Liver Cancer	PTPMT1	HCC	-	Knockout and knockdown	lentiCas9-Blast vector	CRISPR-Cas9 knockdown library screening revealed PTPMT1 in the production of cardiolipin as critical to survival in hypoxia in liver cancer.	[77]
Liver cancer	Pten, Rb1, and Ctnnb1	-	Mice	-	px459 V2.0 vector	CRISPR/Cas9-induced Liver cancer mouse model: Longitudinal imaging of liver cancer Using MicroCT and nanoparticle contrasting agents.	[78]
Liver Cancer	Traf3	HepG2	-	Knockout	Lentiviral	The CRISPR/Cas9 method improved HepG2 cell proliferation, migration, and invasion and provided a helpful tool for researching Traf3 function and mechanism.	[79]
Liver cancer	ARID1A,	HCC	Pig	Knockout	-	CRISPR/Cas9 editing of pig liver cancer cells to create genetically customized cancer cells.	[80]

**Table 2:** The efficacy of CRISPR-based cancer therapeutics in several different clinical studies

Clinical Trials Identification	Country	Developer	Disease	Number of participants	Target Gene/modification	Delivery	Study Phase	Estimated Study Completion Date	References
NCT03655678	Canada, Europe	Vertex Pharmaceuticals Incorporated	Beta Thalassemia	45	Analogous CD34+ HSPCs that have been reprogrammed at the BCL11A gene enhancer.	Ribonucleoprotein electroporation	Phase 2 Phase 3	August 2024	[81, 82]
NCT03728322	Unknown	Allife Medical Science and Technology Co., Ltd.	Beta Thalassemia	12	HBB gene is corrected in iHSCs patient-specific	Unspecified	Early Phase 1	January 31, 2021	-
NCT04205435	China	Bioray Laboratories	Beta Thalassemia Major	12	Autologous hematopoietic stem cells gene-edited with β-globin restoration.	Unspecified	Phase 1 Phase 2	December 1, 2023	[81]
NCT04211480	China	Bioray Laboratories	Thalassemia Major	12	Gene-edited autologous hematopoietic stem cells with γ-globin expression	Unspecified	/	June 1, 2023	-
NCT03745287	US, Europe	Vertex Pharmaceuticals Incorporated	Severe Sickle cell disease	45	Autologous CD34+ HSPCs modified at the enhancer of the BCL11A gene	Ribonucleoprotein electroporation	Phase 1 Phase 2	October 2024	[81–83]
NCT04774536	US	Mark Walters, MD	Sickle cell disease	9	Autologous CD34+ HSPCs modification	Ribonucleoprotein	Phase 1 Phase 2	December 1, 2026	-
NCT04037566	China	Xijing Hospital	1. Leukemia Lymphocytic Acute (All) Refractory 2. Lymphoma, B-Cell	40	CRISPR gene-edited to eliminate endogenous HPK1(XYF19 CAR-T cell)	Ribonucleoprotein electroporation	Phase 1	August 2024	-
NCT03398967	China	Chinese PLA General Hospital	(1) B cell leukemia, (2) B cell lymphoma	80	Allogeneic CD11 and CD20/22 directed CAR T cells	Unspecified	Phase1 Phase2	May 20, 2022	-
NCT03166878	China	Chinese PLA General Hospital	(1) B cell leukemia, (2) B cell lymphoma	80	Allogeneic CD19-directed CAR T cells; TCR and B2 M disruption	RNA electroporation	Phase1 Phase2	May 21, 2022	-
NCT03690011	US	Baylor College of Medicine	1. T-cell Acute Lymphoblastic Leukemia 2. T-cell Acute Lymphoblastic Lymphoma 3. T-non-Hodgkin Lymphoma	21	Anti-CD7 CAR T cells, CD7 KO	Unspecified	Phase1	May1, 2038	-
NCT04637763	US	Caribou Biosciences, Inc	1. Lymphoma, Non-Hodgkin 2. Relapsed Non-Hodgkin Lymphoma 3. Refractory B-Cell Non-Hodgkin Lymphoma 4. Non-Hodgkin Lymphoma 5. Lymphoma 6. B Cell Lymphoma 7. B Cell Non-Hodgkin’s Lymphoma	50	CRISPR-edited allogeneic CAR-T cell therapy- CRISPR-edited targeting CD19 (CB-010)	Unspecified	Phase 1	September 2025	-
NCT03545815	China	Chinese PLA General Hospital	Solid Tumor, Adult	10	PD-1 and TCR KO anti-mesothelin CAR T cells	Unspecified	Phase 1	December 30, 2020	-
NCT03747965	China	Chinese PLA General Hospital	Solid Tumor, Adult	10	Mesothelin-directed CAR-T cells	Unspecified	Phase 1	May 2020	-
NCT03081715	China	Hangzhou Cancer Hospital	Advanced Esophageal Cancer	16	PD-1 KO T Cells	Unspecified	-	Completed	[84–87]
NCT02793856	China	Sichuan University	Metastatic Non-small Cell Lung Cancer	12	PD-1 KO T Cells	Unspecified	-	Completed (March 17, 2020)	[85–92]
NCT03044743	China	Yang Yang, Nanjing University Medical School	1. Stage IV Gastric Carcinoma 2. Stage IV Nasopharyngeal Carcinoma 3. T-Cell Lymphoma Stage IV 4. Stage IV Adult Hodgkin Lymphoma 5. Stage IV Diffuse Large B-Cell Lymphoma	20	PD Knockout EBV-CTL	Unspecified	Phase 1 Phase 2	March 2022	[93–98]
NCT04426669	US, UK	Intima Bioscience, Inc	Gastrointestinal cancers	20	Gene Encoding autologous CISH-inactivated TILs	Unspecified	Phase 1 Phase 2	October 2022	-
NCT03872479	US	Editas Medicine, Inc.	1. Blindness 2. Leber Congenital Amaurosis 10 3. Eye Diseases	18	Eliminate the mutation on the CEP290 gene	AAV	Phase 1 Phase 2	March 22, 2024	[99]
NCT04560790	China	Shanghai BDgene Co., Ltd	1. Viral Keratitis 2. Blindness Eye 3. Herpes Simplex Virus Infection 4. Cornea	6	BD11 CRISPR/Cas9 mRNA Instantaneous Gene Editing	Unspecified	Phase 1 Phase 2	May 2022	-
NCT03332030	US	Roger Packer	1. Neurofibromatosis Type 1 2. Tumors of the Central Nervous System	20	Fix NF1 mutation allele	Unspecified	-	July 1, 2025	-
NCT03164135	China	Affiliated Hospital to Academy of Military Medical Sciences	HIV-1-infection	5	CD34+ hematopoietic stem/progenitor cells From donor are treated with CRISPR/Cas9 targeting CCR5 gene	Unspecified	Not Applicable	May 20, 2021	[100]
NCT04244656	Swiss/American	CRISPR Therapeutics AG	Multiple Myeloma	80	CTX120 B-cell maturation antigen (BCMA)-directed T-cell immunotherapy comprised of allogeneic T cells genetically modified ex vivo	Unspecified	Phase1	January 2027	[101]
NCT04035434	Swiss/American	CRISPR Therapeutics AG	1. B-cell Malignancy 2. Non-Hodgkin Lymphoma 3. B-cell Lymphoma 4. Adult B Cell ALL	143	CTX110 (CD19-directed T-cell immunotherapy comprised of allogeneic T cells genetically modified ex vivo	Unspecified	Phase1	August 2026	-
NCT04767308	China	Huazhong University of Science and Technology	1. CD5+ Relapsed/Refractory hematopoietic malignancies 2. Chronic lymphocytic leukemia (CLL) 3. Mantle Cell Lymphoma (MCL) 4. Diffuse large B-cell lymphoma (DLBCL) 5. Follicular lymphoma (FL) 6. Peripheral T-cell lymphomas (PTCL)	18	CT125A chimeric antigen receptor (CAR) T cells	Unspecified	Early phase 1	December 2023	-
NCT04417764	China	Central South University	Advanced Hepatocellular Carcinoma	10	PD-1 KO T Cells	Unspecified	Phase 1	December 31, 2021	-
NCT03525652		The First Affiliated Hospital of Guangdong Pharmaceutical University	Prostate Cancer	30	PD-1 KO T Cells	Unspecified	Phase 1 Phase 2	August 30, 2021	-
NCT04601051		Intellia Therapeutics	Hereditary Transthyretin Amyloidosis	38	NTLA-2001	lipid nanoparticles	Phase 1	March 2024	[102, 103]

### Challenges of CRISPR/Cas9

**Fig. 5:**
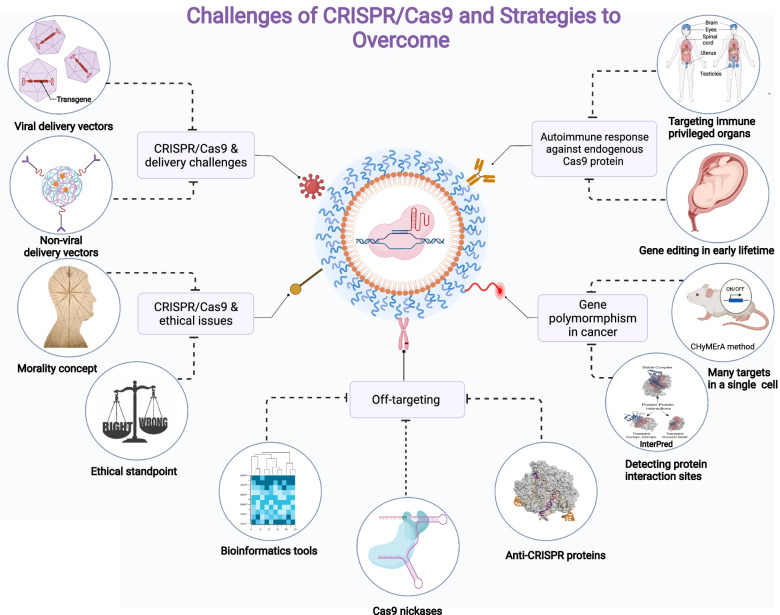
Challenges and overcoming strategies of CRISPR/Cas9. Immunogenicity, off-targeting, polymorphism, delivery technique, and ethical issues are main limitations, difficulties; and challenges of the CRISPR/Cas9 system in clinical trials and its recent discovery and usage in humans

Even though the previous explanation suggests that CRISPR/Cas9 is a promising approach, this editing system still has a number of limitations and risks that make it challenging to use in clinical trials due to its recent discovery and use in humans. Immunogenicity, off-targeting, polymorphism, delivery method, and ethics are only several major concerns with the CRISPR/Cas9 system highlighted with the list of strategies that has been developed and can be used to overcome those limitations (Fig. 5).

### Autoimmune response against endogenous Cas9 protein

The Cas9 protein is one of the three main components of the structure of the CRISPR system, and it has a fundamental role in binding double-stranded DNA, paired with the mRNA guide, and cutting it at a specific site, expressly 3 bases before the PAM sequence [104].This protein derives from *Streptococcus pyogenes*, a bacterium that is the cause of many common infections in humans. It is recognized by the body as an antigen, developing an immune response against it [105]. Similarly, the existence of a pre-existing immune response to the homologous Cas9 protein in *Staphylococcus aureus* has been reported [106]. Indeed, both *Staphylococcus aureus* and *Streptococcus pyogenes*, from which the main Cas9 proteins are obtained, SaCas9 and SpCas9, have infected humans for a long time [106]. Thus, the human immune system recognizes these proteins as foreign and develops an immune response against them upon injection, which leads to fast degradation of the Cas9 protein, preventing it from performing the gene-editing function [107].

### Strategies to overcome immunogenicity

**Fig. 6:**
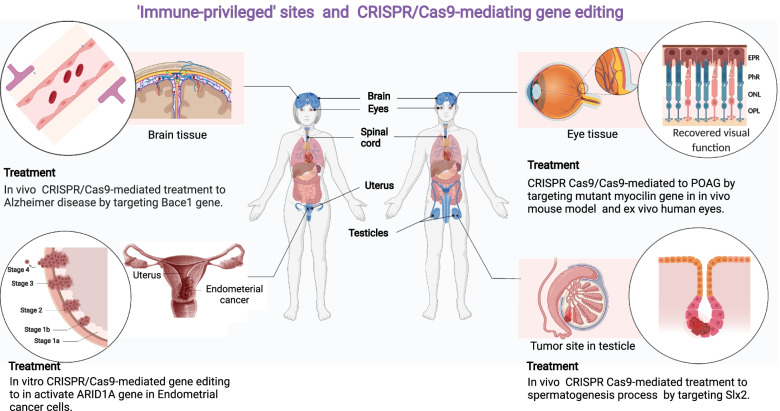
‘Immune-privileged’ sites and CRISPR/Cas9-mediating gene editing. Implementing the CRISPR Cas system for gene editing early in person’s life; and targeting immune-privileged organs are all attempts to overcome the limitations provided by immunogenicity against Cas9

Several strategies have been proposed to overcome limits posed by immunogenicity against Cas9. Here, we are giving an overview of the main ones offered; (i) implementing the CRISPR/Cas system for gene editing early in a lifetime; (ii) targeting immune-privileged organs (Fig. 6).

### Gene editing in early lifetime

Even before birth, various types of disease can be detected in children, and preventing or treating those diseases will save thousands of lives worldwide. The CRISPR/Cas system has been successfully applied in treating various types of inherited diseases in children, such as cystic fibrosis, thalassemia, and sickle cell anemia, Mucopolysaccharidosis type IVA [108–113]. Furthermore, CRISPR/Cas9 can inhibit different molecular pathways of various common types of cancer in children, such as neuroblastoma and lymphoma [114, 115]. Moreover, treating these defects by CRISPR Cas system after diagnosed can be done before the infant is immunized with anti-Cas protein.

### Targeting immune-privileged organs

Another practical approach to overcoming the risk of autoimmune disease is gene editing by CRISPR Cas9 techniques in those organs recognized as immune-privileged organs. An Immune privileged organ can be defined as a site in the body where a graft tissue can be implanted without being rejected by the organism due to an immunological reaction formed against it [116]. Examples of immune privilege organs are eyes [117], brain [118], placenta, fetus [119], and testicles [120].

Many congenital eye disorders lead to blindness and other defects in the eyes, such as Leber congenital amaurosis type 10, retinal dystrophy caused by a mutation in the CEP290 [121]. Fortunately, many studies proved that the eyes are one of the immune-privileged organs that can successfully imply CRISPR Cas9 on it and edit a particular mutation there [122]. For example, Jain et al. employed CRISPR-Cas9 genome editing in human TM cells and in a POAG animal model to reduce the expression of mutant MYOC, resulting in a reduction in the stress on the ER [123].

CRISPR offers an excellent opportunity for scientists to reach high gene editing efficiency in fetuses and embryos, as the immune system has not yet reached maturity. Nevertheless, Because of the substantial danger of embryo off-targeting associated with its use *in vivo*, it is illegal in many countries. For example, CRISPR/Cas9’s off-targeting rate was 16% in a study aiming to target the POU5F1 gene in embryos [124]. Correspondingly, due to the cleavage of both alleles, off-target cleavage of Cas9 causes chromosomal loss and hemizygous indels [125]. These findings show that chromosomal content can be manipulated. Still, it requires other skills and strategies to reduce the high risk of off-targeting and loss of DNA fragments.

Additionally, testicles are another immuno-privileged organ that the gene editor can target to correct the mutated genes and deactivate oncogene in cancer patients [120]. These genes can be identified and reverted to their normal function through CRISPR Cas system. Sun et al. found that male fertility genes in mice can be dispensable for further fecundity by knocked out through CRISPR/Cas9 [126]. Furthermore, in mice, CRISPR/Cas9-mediated gene editing uncovered 30 testis-enriched genes not required for male fertility [127].

Likewise, brain is another immune privileged organ, and several studies were performed *in vivo* without immune tolerance. Normalized FMR-1 gene expression was achieved by CRISPR/Cas9-mediated deletion of the CGG repeat in hiPSCs from fragile X syndrome patients, a change that was sustained even after differentiation into neural progenitor cells (NPCs) and mature neurons; in addition, hypermethylation of the CpG sites upstream of FMR-1 was reversed [128].

### Off-targeting

Another main concern about using CRISPR/Cas9 in recent years is having a high number of off-targeting [129–131]. When implying the CRISPR Cas9 system in a complex genomic species such as mammalians, the gRNA might bring to a wrong target due to similarities within the genome, which may lead to further mutations being introduced in undesired genomic locations [132]. In recent years, many bioinformatics tools have been developed to help predict and reduce off-target modifications. These should be further improved to enable researchers to use them effectively in the development of new therapies.

### Strategies to overcome off-targeting

The main strategies that have been successfully performed in previous studies can be classified into three main groups; (I) bioinformatics tools to design more accurate gRNA and predict off-targeting; (II) use of Cas9 nickases; (III) add anti-CRISPR proteins.

### Bioinformatics tools

Bioinformatics tools play a crucial role in analyzing, predicting, and determining the CRISPR Cas system. Bioinformatics tools allowed Francisco Mojica to discover that the system previously found in bacteria also existed in archaea [133]. Further, bioinformatics tools help scientists design more efficient gRNAs, detect the accurate editing site within the whole genome, and evade off-targeting percentage probability (Table 3) [134]. Studies have shown that the gRNA is responsible for most of the off-targeting [135]. For example, many studies have shown a direct correlation between gRNA length and the number of off-targeting; thus, finding the perfect size of the gRNA is essential to reduce the off-targeting probability [136]. Such as reducing the length of gRNA to less than 20 nucleotides have a significant role in lowering off-targeting by about 5000 folds in the same efficiency of the longer gRNA [34, 122]. According to another study, most of the mismatches occur within the last three nucleotides placed at the opposite side of the PAM sequence, thus removing these nucleotides and maintaining the length of gRNA about 17 nucleotides crucial role in the reduction of off-targeting [137]. On the other hand, gRNAs shorter than 15 base pairs are not safe as they would lose the specificity and could not bind the right target inside the nucleus [138].

**Table 3:** Online bioinformatics tools detect the accurate editing site within the whole genome and evade off-targeting percentage probability

Tool name	Description	Input	Output	Maximum mismatches allowed	Supported nucleases	PAM sequence	References
CRISPResso2	Genome editing and interpretation of amplicon sequencing	1. Editing of tool specification. 2. Input sequences 3. Amplicon sequence, sgRNA sequence	1. indel sizes and positions 2. HDR/NHEJ frequency 3. sequence alignment with reference 4. allele-specific quantification	-	Cas9 Cpf1	NGS	(139)
Cas-Analyzer	Genome editing and programmable nucleases	1. Fastq 2. gzip-compressed	1. indel sizes and positions 2. HDR/NHEJ frequency 3. sequence alignment with reference	up to a 1-nt	SpCas9, StCas9, NmCas9, SaCas9, CjCas9, AsCpf1/LbCpf1, paired nucleases: ZFNs, TALENs, Cas9 nickases, dCas9-FokI	NGS	[140]
CRISPR-GA	Quantification of the edited site then analysis of the different alterations.	Paired-end reads	1. indel sizes and positions 2. HDR/NHEJ frequency	< 20	Cas9	NGS	[141]
TIDE/TIDER	Identification of major induced mutations in the editing site using specially developed decomposition algorithm	DNA from a pool of cells treated with RGEN Cas9) and a character string representing the sgRNA sequence (20 nt)	1. indel sizes and positions 2. HDR/NHEJ frequency	∼1%	SpCas9, SaCas9, St1Cas9, NmCas9, AsCpf1, FnCpf1, LbCpf1	Sanger sequencing	[142] [143]
CRISPR-ERA	Analyze gene editing and gene regulation	Sequence starts with N20NGG	1. gRNA design 2. E score (efficacy score) 3. S score ( specificity score) 4. E+S score: the sum of efficacy score and Specificity score.	3	CRISPR/Cas9	NGG NAG	[144]
CRISPRseek	Using various tools for the CRISPR editing including Base Editors and the Prime Editor for input target sequences,	The RNAs sequence is annotated with a total score of the top5 and topN off-targets and Cas9	1. gRNA design 2. off-targeting count 3. score on targeting 4. find Spacer	4	User customizable	NGG NAG	[145]
CHOPCHOP v3	Web tool for selecting alternative transcription of RNA using CRISPER-CAS13	gene name, genomic coordinates, or a pasted sequence (including RefSeq and ENSEMBL gene IDs)	1. gRNA design, Off-targeting 2. GC content (%) 3. number of self-complementary 4. efficiency	-	CRISPR effector (e.g., Cas9, CasX, or Cas13)	NGG	[146]
E-CRISP	Specific Algorithm used to target any nucleotide sequence ranging from single exons to entire genomes	FASTA	1. gRNA design (for various targeting purposes) 2. gene annotation filtering 3. off-targeting analysis. 4. image for (genomic context, restriction site, TSS, strop, and start Condon)	-	genomic context (e.g., exons, transcripts, CpG islands)	NGG NAG	[147]
CRISPy-web	Design sgRNAs	API antiSMASH	gRNA design, Target Site selection	3	Cas9	NGG	[148]
CRISPR-P 2.0	Genome editing in plant	Gene name, ID, position, and sequence	1. on-target score 2. off-target score 3. GC content 4. restriction endonuclease site	-	Cas9	NGG NAG	[149]
COSMID	Validation and identification of off-target sequence	FetchGWI search program	Off-target score GC content (%)	3	CRISPR Off-target Sites with Mismatches, Insertions, and Deletions	NGG, NAG, NRG	[150]
WU-CRISPR	Gene editing and detection of CRISPR/Cas9 Knockout	Gene Sequence in FASTA format	1. gRNA sequence 2. potency score 3. off-target status 4. BLAST alignment 5. coding sequence	-		NGG,	[151]
Cas-Designer	Selecting all RGEN targets via Microhomology-predictor	FASTA	1. RGENs 2. Cas-OFFinder 3. Cas-Designer 4. Cas-Database.	0-10	NGG, NRG, NNAGAAW, NNNNGMTT	It depends on the Cas protein.	[152] [153]
CRISPR MultiTargeter	Web program to detect the High identical site in multiple genes	FASTA	Multiple sequence Alignment	0-24	1. SpCas9 (PAM ‘NGG’), 2. StCas9 (PAM ‘NNAGAAW’), 3. NmCas9 (PAM ‘NNNNGMTT’)	NGG	[154]

### Cas9 nickases

**Fig. 7:**
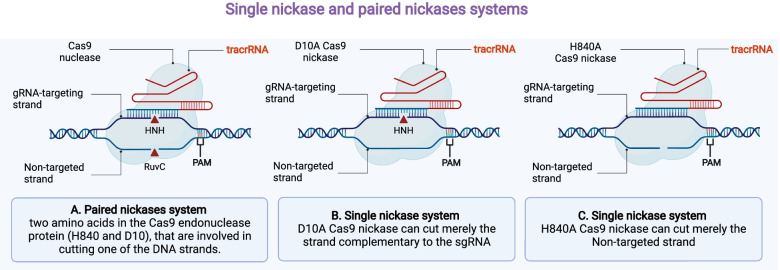
Nickase systems consisting of one or two nickases. H840 and D10 are two amino acids found in the Cas9 endonuclease protein that are involved in the cutting of one DNA strand by the enzyme. The RuvC domain contains the amino acid H840, while the HNH domain has the amino acid D10. The non-targeted strand is cleaved by Cas9 H840A, while the gRNA-targeting strand is cleaved by Cas9 D10A. Cas9 can only cut the strand complementary to the gRNA in a single nickase; however, a pair of sgRNA-Cas9n complexes can nick both strands at once (paired nickases). Additional concerns for gRNA design when using paired nickases include creating a 5’ overhang, the spacing between the two gRNAs, and the relative position of the two gRNA target sites

Another practical approach to reducing the number of off-targeting is mutating in one nuclease domain in just one strand of the DNA by CRISPR nickase, which crucial to create nick that quickly repaired in the cells nickase [155]. Cas9 nickase has a different breaking mechanism than the normal Cas9 protein; in particular, it breaks down just one strand of the DNA, and they use double adjacent gRNAs rather than sgRNAs (Fig. 7). Therefore, editing genes by using Cas9 nickase reduces further damage in the target DNA, and it has a significant role in reducing the number of off-targeting [156]. Furthermore, it was shown that paired nicking could reduce the risk of off-targeting by 50 to 1500 folds in cell lines, and in mouse zygotes, it allows the gene knockout without any effect on cleavage efficiency [155].

### Anti-CRISPR proteins

Inactivation of Cas9 protein after targeting its site may also reduce the number of off-targeting [134]. It has been proven that the number of off-targeting is correlatively increased as long as the Cas9 protein is expressed in the human tissue culture [137]. Deactivation of Cas9 protein can be obtained through using anti-CRISPR proteins (Acr) [157]. Acr proteins are produced in both bacterial and human cells and allow to disable CRISPR function [158]. Moreover, more than 50 anti-CRISPR proteins have been discovered so far, synthesized by viruses as a defense system against prokaryotic cells [159]. The first Acr protein discovered that deactivates the CRISPR type I system in *P. aeruginosa*, while the other Acr proteins can act on different types of CRISPR, such as types II, III, and V [31]. Acr proteins are about 52 to 333 amino acids, meaning they are tiny molecules and diverse with no sequence overlap with other proteins [159, 160]. Also, each Acr protein has a specific and unique sequence free of conserved sequences, which increases their diversity [161]. Having a small size and a unique genomic sequence make the recognition of Acr difficult by standard homology-based methods. Therefore, these proteins can target their aimed sequences before being recognized. Furthermore, using a different mechanism is one of the successful keys used by Acr to deactivate the CRISPR/Cas9 system [162]. For example, AcrIIA4 binds to both Cas9 and sgRNA rather than binding with just one of them [161]. The efficiency of Acr depends on three main mechanisms, which are the crRNA concentration, DNA binding obstruction, and DNA cleavage inhibition [163]. When the viral genome is injected into the phage, its Acr proteins in a small concentration make the host cells immunosuppression and prepare the bacteriophage for future infections by the phase [164]. Conversely, having a high concentration of Acr proteins and vulnerable bacteriophage disables the function of the CRISPR system from the infected bacteria [164]. Moreover, Acr proteins have a stronger binding affinity with CRISPR; thus it is required a small concentration disable the function of the CRISPR system. On the other hand, anti-CRISPR-associated (Aca) proteins work oppositely to Acr proteins by preventing the transcription of anti-CRISPR proteins [163]. Therefore, the CRISPR system can be improved by using Aca proteins to suppress Acr proteins. Also, the use of Acr proteins that imply phage instead of antibiotics may overcome the issue of drug resistance[165].

### Screening before the treatment

Pre-existing mutations in genes like TP53 and KRAS may raise the risk of additional mutations during CRISPR Cas cancer therapy [166]. And the two primary ways for dealing with this problem are screening before using the CRISPR Cas system and monitoring the patient after injection.

### Polymorphism in cancer

Unlike other genetic diseases such as Duchenne Muscle, Dystrophy, and cystic fibrosis, cancer relies on several mutations [167–170]. Moreover, dysregulation of the multiple genes leads to cancer most of the time. For example, mutations happen in approximately 190 codons in the human TP53 gene, and around 25% of the mutations occur in eight codons [171]. Hence, editing a single mutated nucleotide is not enough in most cases that are widely performed in gene therapy [170]. Correcting mutated nucleotide by knocking-in is much more challenging in CRISPR Cas9 since it is more precise than knocking out, which creates alterations, as knocking in, all of the cancer-causing genes takes longer and needs multi-guide RNA [172]. However, by CRISPR Cas9, knocking in is potentially helpful in many ways, such as studying particular gene variation to find out the gene regulation [172].

**Fig. 8:**
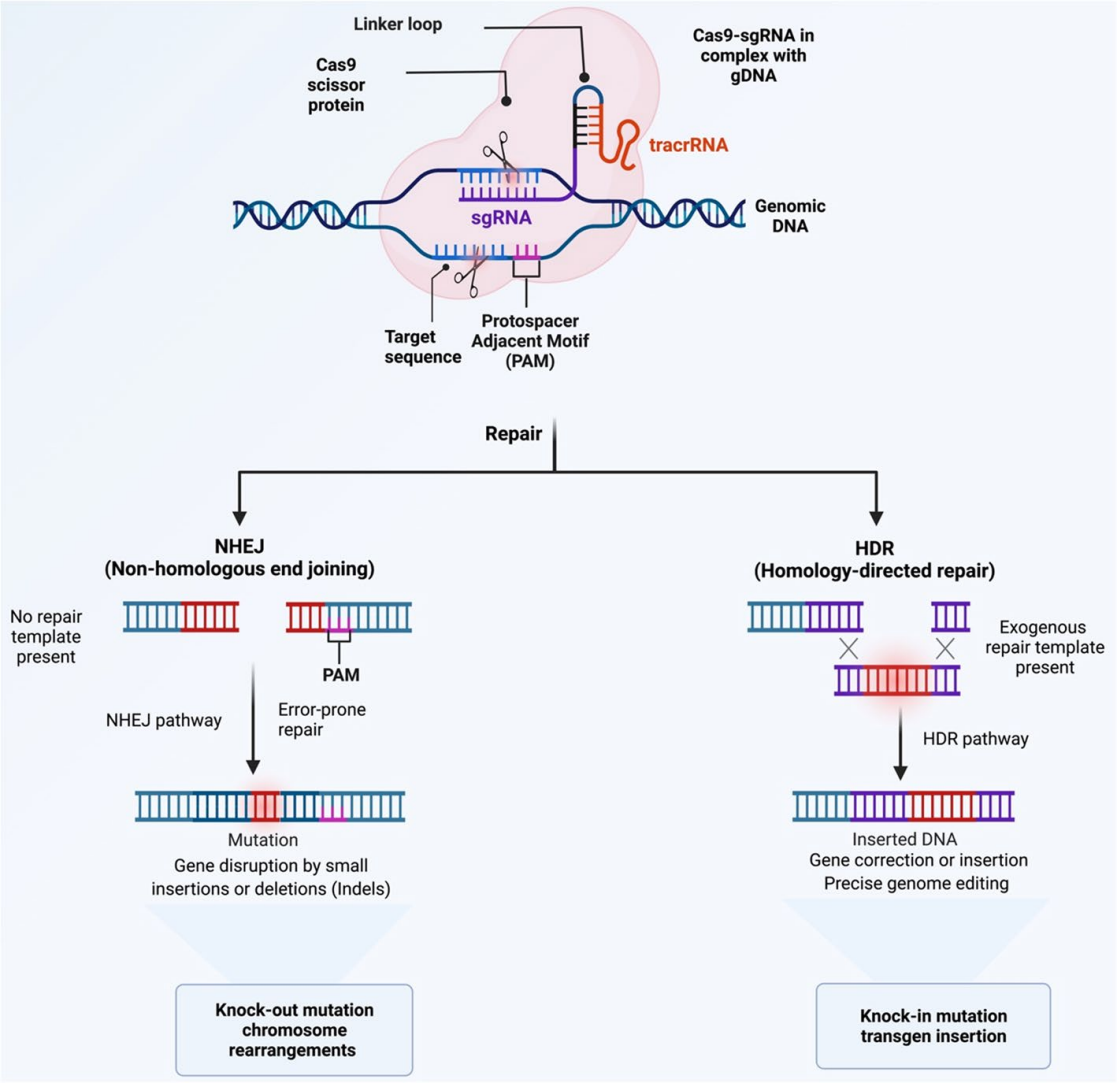
Mechanisms of DNA repair outcomes of genome editing. Typically, DNA double-strand (ds) breaks caused by CRISPR/Cas9 are repaired via either homology-directed repair (HDR) or non-homologous end joining (NHEJ), depending on the circumstances. Exogenous ‘repair templates’ can be introduced into the genome by HDR, whereas NHEJ creates random insertions and deletions (indels) that can disrupt coding areas or catalyse genome rearrangements. The preference for HDR or NHEJ after DNA damage can be increased by small compounds that interfere with each system and so bias the cell toward one or the other after DNA damage

Correcting or editing the mutated nucleotides of tumor suppressor genes is one of the approaches that should be thought about to obtain the desired result in cancer therapy by knock-in in the mutated gene (Fig. 8). CRISPR/Cas9 technology targeted these tumor-suppressor genes to inhibit or reduce tumorigenesis by restoring the activities of tumor-suppressor genes [34]. However, as in cancer, there is plenty of mutations in tumor-suppressor genes, it requires a higher number of gRNA, and there is a higher risk of off-targeting. On the other hand, the CRISPR Cas system can disrupt the nucleotides located in the active site of the protein to suppress the activity of oncogenes, such as KRAS in pancreatic cancer and ATM in neuroendocrine cancer by deleting their inactivation sequences (Table 1) [34, 173]. On the other side, in TNBC cells, the deactivation of CXCR7 and the co-knockout of CXCR4 and CXCR7 have been shown to inhibit the expression of oncogenes and may have a potential target in TNBC treatment [52]. For the Cas system to be effective in knocking out oncogenes, the proper gRNA must be designed to target the binding site of oncogenes and prevent protein-protein interaction, which is an essential step in the molecular pathway of cancer progression [52].

### Strategies

We described two primary solutions for managing polymorphism issues: CHyMErA and bioinformatics techniques to investigate the protein interaction site and forecast the results.

### Performing CHyMErA

**Fig. 9:**
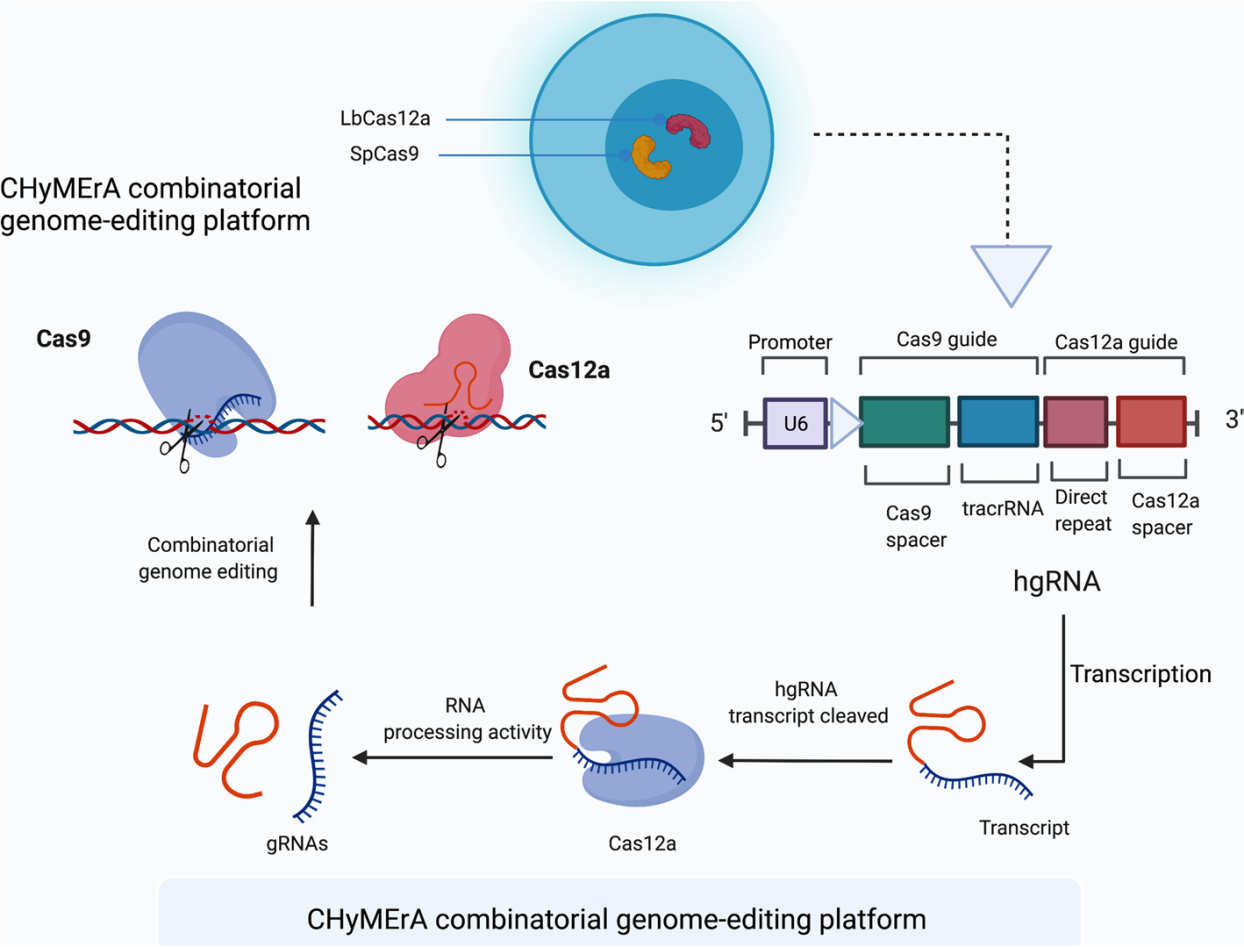
DNA editing platform CHyMErA is a combinatorial system. Cell lines harbouring nuclear SpCas9 and LbCas12a, as well as a hgRNA expression cassette, provide the basis of the CHyMErA system. Cas12a gRNAs are fused with Cas9 and expressed under a single U6 promoter in hgRNAs. This process is completed by Cas12a, which identifies the direct repeat sequence and cuts upstream of it to release functional Cas9 and Cas12 gRNAs that can be loaded onto their respective nucleases for directed combinatorial genome editing

To edit many targets in a single mammalian cell, CRISPR may be utilized with various kinds and procedures, such as the CHyMErA (Cas hybrid for multiplexed editing and screening applications) method. CHyMErA depends upon two Cas proteins, Cas9 and Cas12a nucleases, rather than just the standard CRISPR/Cas9 gene editing (Fig. 9) [174]. Exons may be deleted using CHyMErA, which is helpful for the high deletion of gene sequences. As a result, employing CHyMErA to target multiple sites is one of the novel approaches to overcoming cancer polymorphism [175].

### Detecting protein interaction site

Bioinformatics tools can have a crucial role in predicting and obtaining the desired results in knocking out. For example, different databases can be used for finding out the interaction site of the proteins, such as InterPred [176]. By using this platform, amino acids located in the active site of the proteins can be detected, and then gRNA is designed based on it. Moreover, different databases can be used for predicting the result of CRISPR knockout (Table 3).

In conclusion, knock-in and Knock-out for oncogenes and tumor suppressor genes are critical in gene editing using CRISPR Cas9. However, knocking in to edit a particular nucleotide should be performed more precisely. Indeed, having more than one mutation in cancer cells required performing other techniques such as ChyMErA, which can target multi targets by binding two cutting DNA enzymes Cas9 and Cas12a. On the other hand, creating one mutation in the protein active site of the oncogenes is enough to suppress its role, so performing Knocking out in cancer therapy to suppress oncogene is much more practical.

### The delivery challenges

Choosing a proper safe, and precise delivery technique to carry the CRISPR system into the tumor site, especially *in vivo*, and targeting the right sequence inside the nucleus is another challenge that should be considered. CRISPR/Cas9 technologies are delivered through different approaches, such as viral, physical, and extracellular vesicle-base system delivery techniques [177]. Additionally, each method is used for a specific purpose and has its limitations. Thus, the main challenges while choosing the right vector are packaging, delivery, and targeting the right site [178, 179]. For example, viral vectors are used widely in both *in vivo* and *in vitro*, but it has many limitations, such as immune response and insertional limitation [177]. For instance, after implying viral vector *in vivo*, it exposes continuously for a long time and increases the risk of mutations and off-targeting [180].

### Viral delivery vectors

Adeno-associated viruses (AAV) such as adenovirus and lentivirus have been utilized successfully in other research *in vivo* [181] and they do not cause any other diseases in humans, only a very few immune responses [178, 182]. On the other hand, the main disadvantage of AAV has a tiny packaging size, so more than one AAV is required to carry all the CRISPR systems such as gRNA and Cas protein [182]. Additionally, the maximum size that a single AAV vector can deliver is about 4.7 kbp, while the genomic size of SpCas9 alone is around 4.3 kbp [178]. Thus, more than one victor is necessary to hold all the systems.

### Non-viral delivery vectors

Besides that, non-viral delivery vectors are another approach, such as lipid nanoparticles and inorganic nanoparticles [183]. In addition, a non-viral delivery approach, like nanoparticle-based delivery, allow for more frequent administration of gene therapy with lower risk of immunogenicity, less exposure to nuclease and more accurate targeting [183, 184]. Furthermore, non-viral vectors have a greater capacity than viral vectors without integrating in the carried genome [185]. On the other hand, extracellular vesicle-based systems have been utilized in both *in vivo* and *in vitro* systems successfully and, compared to the other system, are safer and cheaper [186]. Another challenge is the delivery and efficiency percentage of the CRISPR system into the targeted size, especially in cancer therapy with total editing efficiency [178]. And according to Yin et al. (2014), the total delivery efficiency is 1 out of 250 in targeting liver cells using hydrodynamic injection [187] (Table 4).

### Strategies

One of the effective ways to overcome the packaging challenge is splitting the Cas9 protein into two AAV (AAV-split-Cas9) vectors instead of one [188]. As previously explained, large size vectors increase the risk of off-targeting and mutation [189]. As a result, employing a smaller Cas9 protein and splicing it into two AAV vectors is critical for reducing off-targeting and increasing delivery efficiency [190]. Another option that can be used to reduce the risk of off-targeting associated with delivery techniques is the use of ribonucleoprotein (RNP) complexes, such as recombinant CRISPR-Cpf1 Ribonucleoprotein (CRISPR-Cpf1-RNP) suppressed off-target activity in mouse cells [191]. Furthermore, according to their results, Mout and his colleagues applied Cas9-RNP methods, which efficacy around 95% in cultured cells [192]. This approach also degrades after 24 to 48 h of injection [182] (Table 4). Thus, the risk of further mutations and off-targeting that occur due to the continuous expression of viral vectors is reduced significantly [177] .

**Table 4:** Summary of accomplishments and challenges for viral and non-viral delivery approach of CRISPR Cas system

Therapeutic genome editing approaches	Delivery methods	Targets or disease	Genome editing accomplishments	Carrying capacity	Challenges	Strategies	References
Viral vector	Adenovirus	T cells	CCR5 knockout is in clinical trials	37 Kb	In vivo, immunogenicity is a major restraint.	Targeting immune privilege organs such as eyes, brain, uterus.	[193, 194]
	AAV in vitro	T cells and HSCs	High genome editing rate as a donor; can be paired with non-viral nuclease delivery	4.7 kb	HDR donor size is limited by vector carrying capability.	It is possible to generate donor templates for HDR-mediated methods by infecting AAV vectors with a ssDNA vector genome	[195–197]
	AAV in vivo	brain, retina, Liver, heart, muscle	In animal models, knockouts and HDR have been produced; this can be used with non-viral nuclease delivery.	4.7 kb	1. There are still issues with delivery efficiency and preexisting immunity to natural serotypes. 2. Exposes continuously for a long time after implying in vivo and increase risk of off-targeting 3. Having a small packaging size, 4.7 kbp, while the genomic size of SpCas9 alone is around 4.3 kbp 4. Hepatoxicity	1.a.To eliminate pre-existing immunity to AAV, it can be employed alone or in conjunction with other approaches. 1.b. Targeting immune privilege organs such as eyes, brain. - 3.a. splicing the Cas9 protein into two AAV vector (AAV-split-Cas9) can be performed. 3.b. Choosing a smaller size of Cas9 protein such as SpCas9 which is 1 kilo base shorter. -	[183, 196, 198, 199, 178, 183, 188, 200]
	Lentiviral vector	In retina and in vitro	Lentivirus with integrase defects utilized as a donor	8 kb	The de novo expression of a protein lacking in the host may result in immune responses leading to the clearance of the transduced cells and the formation of antibodies that inhibit the activity of secreted factors	Cyclosporine, tacrolimus, and cyclophosphamide can inhibit the synthesis and secretion of cytokines and prevent the activation and proliferation of T cells	[201–203]
Non-viral vector	Electroporation	In vitro: T cells, HSCs; in vivo: muscle and kidney	High genome editing efficiency in cells difficult to transfect	-	1.Only feasible in ex vivo applications; in vivo electroporation is limited to mice, unclear if possible in humans	1.a. combining the CRISPR/Cas9 system and in utero electroporation is an effective and rapid approach to achieve brain-specific gene knockout in vivo. 1.b. electroporation does not require microinjection skills and can be used to treat 40–50 embryos simultaneously.	[204, 205]
	Lipid-based delivery vehicles	PCSK9, TTR, TMC1	High NHEJ efficiency for hepatocytes and hair cells in vivo. Minimize immunogenicity Reduce off-targeting	-	1.a.Cas9 mRNA may activate TLRs. 1.b. Due to the constant positive charge, these formulations induce toxicity, adverse reactions, and immunogenic responses	1.Lipid nanoparticles (LNPs) based on ionizable cationic lipids were developed to circumvent these restrictions	[200, 206]
	Microinjection	In vivo: zebrafish Caenorhabditis elegans		-	1. Cell damage 2. Only a single cell can be targeted in each injection.	1.To reduce cell damage, a high level of sophistication and manual skills are required. -	[207, 208]
	iTOP		iTOP transduction is effective for intracellular delivery of the Cas9 protein and sgRNAs independently, or direct delivery of RNPs.	-	Lower efficiency in primary cells. Since it is only soluble at high salt concentrations, it is not adequate for in vivo.		[209]

### Ethical issues and CRISPR/Cas9 technology

Human genetic alterations have long been a source of ethical debate; CRISPR/Cas9-mediated genome editing has provided a new perspective. Considering the unpredictability and broad-reaching effects of this technology’s appealing applications, a thorough examination of its ethical and societal implications is required. The conceptions of various members of society, such as the public and religious academics, are fundamental.

### Current ethical standpoint

The application scope of CRISPR/ Cas9 is expanding at an incredible rate. Switching genes on or off to investigate how they work or causing mutations in cells to learn why and how they become malignant, are some of the opportunities it has opened up in molecular biology research. Gene editing can be used to create resistant crops and stronger police dogs, for example [210, 211]. Another highly contentious concept would edit the human genome permanently to eliminate disease-causing mutations or even improve or introduce desired features in offspring by inserting helpful genes and this is debatable [212]. Non-reproductive cell genome modifications are not heritable, whereas germ cell modifications can be passed down to the next generation. As a result, the attractive uses of this approach raise ethical, moral, and safety concerns [213]. Human germline modification using CRISPR/Cas9-based gene editing has raised concerns about threats to human safety and dignity, as well as the potential for genocide. There was an effort to halt human genome research until a national or global agreement on society’s acceptance of this new technology was reached [214].

### Morality concepts

Morality concepts, particularly in biomedicine, are based on empirical research and entail evaluating potential risk-benefit ratios, to maximize the latter while decreasing the former. It is vital to assess the spectrum of conceivable outcomes, the likelihood of each occurring, and the various arguments for the outcomes of any one while making moral decisions. There are at least three major causes for ethical concerns concerning CRISPR genome engineering technology. Concerns have been raised about the power and technical limitations of CRISPR technology in the first concept. These drawbacks include a lack of on-target editing efficiency [215], incomplete editing (mosaicism) [216, 217], and inaccurate on-target or off-target editing [218, 219]. CRISPR experiments with animals and human cell lines have revealed these limitations. Technology, on the other hand, is evolving at a tremendous speed. The second concern is for the transformed species’ long-term survival: if they will be influenced indefinitely and whether the edited genes will be passed down through generations, perhaps influencing them in unanticipated ways.

Making precise predictions regarding the future of a modified creature and estimating potential hazards and advantages may be difficult, if not impossible, given the aforementioned technical constraints and the intricacies of biological systems. As a result, the uncertainty created by these circumstances makes precise risk/benefit assessments difficult, making moral decision-making more difficult [220]. Finally, even if the genome is altered as planned and the necessary functional output is achieved on time, the complicated link between genetic information and biological phenotypes is not fully understood, according to the skeptical viewpoint. As a result, depending on the circumstances, the biological impact of altering a gene in germline and/or somatic cells may be unknown. The intricate regulatory actions of many genes govern many biological features [221]. As a result, “designing” a biological phenotype at the organismal level is difficult, if not impossible.

### Strategies

On the Brightside, it has the potential to make a significant difference in terms of health and wellbeing if used properly [222]. There are several reasons why this technology can be used correctly, although patient safety is one of the most important. One of the most compelling arguments in favor of allowing the use of this technology is the need to protect patients [212]. When germline editing research is applied in a clinical setting to avoid the inheritance of a specific genetic condition, it may alleviate the sorrow and anxiety that parents encounter in the life of the possibility that their child may be born with that genetic disease [214, 223]. Recently, Bioengineer Feng Zhang of MIT and Harvard has modified the Cas9 enzyme to limit mutations outside its target region [224]. Furthermore, the error rate of CRISPR/Cas9 might be further decreased to a safe range if further modifications are introduced [225]. Considering this, CRISPR/Cas9 mediated genome editing safety concerns might be overcome to some extent. Overall, CRISPR/Cas9 technology’s risk profile varies depending on the design. After overcoming some ethical and safety problems, some are approved or predicted to be used soon. On the Brightside, it may significantly improve health and quality of life, but still, it relies on how this technique is used.

## Conclusion and future perspectives

The therapeutic genome editing field has made tremendous progress in recent years, progressing from essential investigation to preclinical development and into human trials, in particular for ex vivo HSC and T cell editing and also for *in vivo* liver genome editing, as a result of the relatively efficient delivery methodologies developed for these systems. However, several considerable challenges need to be addressed before the biomedical promise of genome editing can be fully realized. First, delivery has always been one of the most, if not the most, formidable problems in the gene therapy field. Importantly, HDR and even knockout efficiencies are currently low in many tissues, so higher delivery efficiency is needed to compensate. There should be an international law to ensure that gene editing does not harm humanity, and experiments should be restricted in a health care system.

## Data Availability

The analyzed data sets generated during the study are available from the corresponding author on reasonable request.

## Abbreviations

CRISPR/Cas9: clustered regularly interspaced short palindromic repeats-associated protein9
ZFNs: Zinc-finger nucleases
TALEN: Transcription activator-like effector nucleases
TTTN: T-rich PAMs
tracrRNA: trans-activating crRNA
crRNA: CRISPR RNAs
PD-1: programmed cell death-1
PD-L1: programmed death-ligand 1
MRCL: myxoid/round cell liposarcoma
POAG: Primary open angle glaucoma
hiPSCs: Human induced pluripotent stem cells
NPCs: neural progenitor cells
gRNA: Guide RNA
Acr: anti-CRISPR proteins
CXCR: Chemokine receptors
CHyMErA: Cas hybrid for multiplexed editing and screening applications
AAV: Adeno-associated viruses
RNP: ribonucleoprotein
CRISPR-Cpf1-RNP: CRISPR-Cpf1 Ribonucleoprotein
HDR: homology-directed repair
NHEJ: non-homologous end joining
HSCs: hematopoietic stem cells
